# Adaptive response to electrical pulse stimulation is impaired in FSHD myotubes by DUX4 gene network activation

**DOI:** 10.1038/s41598-025-32385-0

**Published:** 2025-12-17

**Authors:** Xiangduo Kong, Ananya Rajagopal, Skylar Renee Foust, Jonovan Osorio, Ali Mortazavi, Anna Grosberg, Kyoko Yokomori

**Affiliations:** 1 Department of Biological Chemistry, School of Medicine, University of California, Irvine, USA; 2 Department of Biomedical Engineering and the UCI Edwards Lifesciences Foundation Cardiovascular Innovation and Research Center (CIRC), Samueli School of Engineering, University of California, Irvine, USA; 3 Department of Development and Cell Biology, School of Biological Sciences, University of California, Irvine, CA USA; 4 Department of Chemical and Biomolecular Engineering, Center for Complex Biological Systems, Sue and Bill Cross Stem Cell Research Center, and NSF-Simons Center for Multiscale Cell Fate Research, University of California, Irvine, USA

**Keywords:** FSHD, Electrical pulse stimulation, DUX4, LEUTX, musclin/OSTN, Sarcomere, Cell biology, Developmental biology, Neuroscience, Stem cells

## Abstract

Facioscapulohumeral dystrophy (FSHD) is one of the most common muscular dystrophies with no effective treatment. The disease is linked to abnormal derepression of DUX4 embryonic transcription factor in skeletal muscle, but at very low frequency. How this relates to the disease process and the exact nature of functional defects of FSHD muscle cells remain obscure. We used electrical pulse stimulation (EPS) substituting motor neuron activation to perform quantitative structural, functional, and gene expression analyses of in vitro differentiated control and FSHD skeletal myocytes. We found that EPS effectively stimulated muscle contractile gene expression and contractility activation in control cells, which are impaired in FSHD patient cells. This is accompanied by exacerbated organizational differences of sarcomeric striation. Importantly, this FSHD patient cell phenotype was confirmed in engineered mutant cells carrying *D4Z4* repeat contraction and *SMCHD1* mutation. Notably, FSHD patient/mutant cells fail to activate musclin/OSTN, an exercise-responsive muscle-protective myokine. Overexpression of LEUTX, a major downstream DUX4 target, was sufficient to recapitulate this phenotype, indicating that the phenotype is linked to DUX4 gene network activation. The results demonstrate DUX4-induced cell intrinsic functional defects of FSHD muscle cells in adaptive response to electrical stimulation, suggesting the possible activity-stimulated pathological mechanism of FSHD.

**Supplementary Information:**

The online version contains supplementary material available at 10.1038/s41598-025-32385-0.

## Introduction

Facioscapulohumeral dystrophy (FSHD) is an autosomal dominant muscular dystrophy characterized by progressive wasting of facial, shoulder, and upper arm musculature^1^. It is one of the most common muscular dystrophies^2^. The majority of FSHD cases are caused by monoallelic deletion of *D4Z4* repeat sequences at the subtelomeric region of chromosome 4q (4qter *D4Z4*) (FSHD1 (MIM 158900))^1,3^. *D4Z4* is a 3.3 kb macrosatellite repeat containing an open reading frame for the double-homeobox transcription factor *DUX4* gene, and its abnormal reactivation is linked to FSHD pathology^4–7^. There are only 1–10 *D4Z4* repeats in the contracted allele in FSHD1, in contrast to 11 ~ 150 copies in the intact allele. In the rare FSHD2, there is a modest *D4Z4* repeat contraction (8–20 copies). The *SMCHD1* gene is mutated in > 80% of FSHD2 cases (MIM 158901)^8^ as well as in severe cases of FSHD1, indicating that *SMCHD1* is a disease severity modifier gene^9,10^. In both FSHD1 and FSHD2, heterochromatin normally formed at the *D4Z4* repeat regions, containing DNA methylation and histone H3 lysine 9 trimethylation (H3K9me3), is disrupted, which contributes to *DUX4* derepression^11–13^. Only those individuals with poly(A) signal downstream of the last copy of *D4Z4* repeat (permissive haplotype) develop the disease, further suggesting the significance of the functional *DUX4* mRNA production in FSHD pathogenesis^6,14,15^.

Since *D4Z4* repeats and some of the downstream transcription factors are primate-specific, it has not been straightforward to study the disease in model organisms. Patient myocytes are critical resources for FSHD research though their limited availability combined with variable genetic backgrounds and qualities have been a major obstacle. To address the consequences of FSHD mutations in the isogenic background, we recently generated mutant myoblast lines carrying D4Z4 contraction and/or SMCHD1 mutation from healthy human skeletal myoblast line with a permissive haplotype using CRISPR-Cas9^11^. Double mutants harboring both *D4Z4* contraction and *SMCHD1* mutation synergistically upregulate DUX4 target genes compared to *D4Z4* deletion only mutants, further supporting the critical link between robust DUX4 target gene network activation and disease severity^11,16^.

Though DUX4 upregulation is linked to the disease and its overexpression is cytotoxic, it is expressed in less than1% of patient myoblasts and 3–4% of patient myotubes in vitro^6,17–19^. Using single nucleus (sn) RNA-sequencing (RNA-seq) and high-resolution high-throughput spatial transcriptomics (MERFISH), however, we demonstrated that even those seemingly DUX4-negative patient myotubes have undergone transcriptomic changes^20,21^. Hindered expression of muscle-related genes has been reported in FSHD myocytes compared to healthy control in vitro^11,22,23^. However, since FSHD exhibits no developmental defect of muscle, how these gene expression differences are related to functional cellular phenotype of FSHD muscle cells was unclear.

Electrical Pulse Stimulation (EPS) has been used to substitute motor neuron activity as an in vitro cellular exercise model, and it was found to cause changes in muscle protein gene and myokine gene expression as an adaptive response^24–26^. In order to examine the cell intrinsic functional phenotype of FSHD skeletal myocytes, we applied EPS on in vitro differentiated healthy control and FSHD myocytes to compare their cellular phenotype and gene expression changes. We found distinct EPS-induced gene expression changes in control and FSHD cells, accompanied by exacerbated structural defects in patient/mutant myotubes. Notably, we found robust induction of *musclin/OSTN*, a well-known acute exercise-responsive myokine gene, in control cells, which is impaired in patient/mutant cells. Overexpression of LEUTX, a major DUX4 downstream target transcription factor, was sufficient to recapitulate the phenotype. The results reveal that FSHD myocytes are deficient in adaptation to electrostimulated contraction invoked by DUX4 target gene network activation.

## Results

### EPS provokes distinct gene expression changes in control and FSHD myocytes

**Fig. 1:**
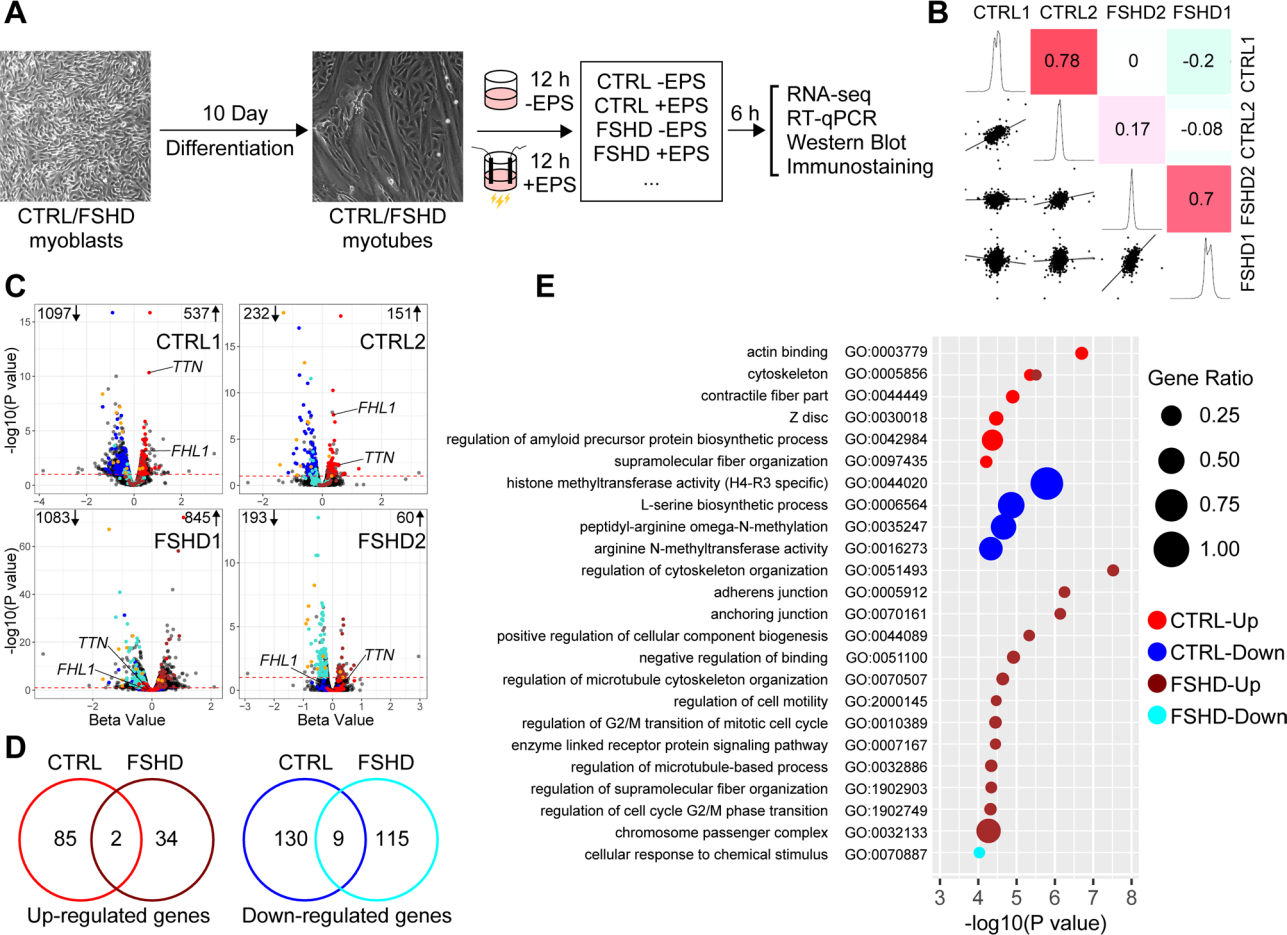
Distinct gene expression changes in response to EPS in differentiated healthy control and FSHD myocytes in vitro. (**A**) Schematic diagram of the experimental setup. Control and FSHD immortalized myoblast cell lines were placed in differentiation media for 10 days with or without EPS for 12 h, followed by samples harvesting 6 h post-EPS. (**B**) Scatterplot matrix of pairwise Spearman correlations and beta value distributions across four cell lines in response to EPS. Beta values of 3,515 genes, which are differentially expressed in at least one cell line after EPS, are analyzed. The diagonal plots show beta value distributions for each cell line. The lower-left panels display scatterplots of beta values between cell lines, while the upper-right panels present Spearman correlation coefficients, visualized using a color gradient from high (red) to low (green). Beta values are calculated through the Wald test based on data from *n* = 3 independent experiments. (**C**) Volcano plots of differentially expressed genes (DEGs) identified in at least one cell line in response to EPS. The fold-change in expression is represented by the Wald test beta value, and significance is indicated by the q-value. Red/blue spots indicate the upregulated/downregulated genes in both Control cell lines but not in both FSHD cell lines, while brown/turquoise spots represent the upregulated/downregulated genes in both FSHD cell lines but not in both Control cell lines. The common DEGs in control and FSHD samples are indicated with orange spots. (**D**) Venn diagram of overlaps of commonly upregulated or downregulated genes in control and FSHD groups. The groups are defined as up-regulated genes in CTRL (upregulated in both Control cell lines, red circle) and FSHD (upregulated in both FSHD cell lines, brown circle), down-regulated genes in CTRL (downregulated in both Control cell lines, blue circle) and FSHD (downregulated in both FSHD cell lines, turquoise circle). (**E**) The bubble plot presents Gene Ontology (GO) enrichment analysis of the DEGs in panel D using Gorilla with the “two unranked lists of genes” option. Bubble colors match the group colors in (D). The x-axis shows log_10_ P-values (threshold of P-value: 0.0001), and bubble size represents the percentage ratio of genes in each term for the respective group as indicated.

In order to investigate the cell intrinsic phenotype of FSHD myocytes, we compared previously characterized two healthy control (CTRL1 and CTRL2) and two representative patient immortalized skeletal myoblast lines (FSHD1 and FSHD2) with robust DUX4 target induction at the late myotube stage (day 11 differentiation) in vitro (Supplemental Fig. S1A)^11^. Presence of DUX4 transcript was also confirmed by RT-qPCR (Supplemental Fig. S1C). Though overall differentiation efficiency is lower in the late stage (~ 16–25%) compared to the early stage (> 90%), individual myotubes in the late stage have more clear sarcomere striation and significantly higher myosin gene expression,^11^ indicating that they are more functionally mature than early myotubes. In both FSHD samples, late myotube differentiation efficiency tends to be lower than controls (Supplemental Figs. S2A and B). These late stage myocytes were subjected to EPS for 12 h and recovered for 6 h (Fig. 1A) as described previously^27^ and analyzed for gene expression using RNA-seq. We found that there are significant global gene expression changes in response to EPS in both control and patient myocytes. Importantly, these EPS-induced gene expression changes correlate well between two controls or between two FSHD cells, but poorly between control and FSHD cells, indicating that FSHD myocytes respond to EPS differently compared to healthy control myocytes (Fig. 1B).

EPS-induced upregulated or downregulated genes (absolute β > 0.2, q-value < 0.1) common in the control samples are largely unchanged in the FSHD samples (Fig. 1C and D, red and dark blue circles, respectively) and vice versa (Fig. 1C and D, brown and light blue circles, respectively) (Supplemental Table S1). GOrilla gene ontology analyses^28^ indicated that muscle contractility and sarcomere organization are uniquely associated with control-upregulated genes (Fig. 1E, red), but unchanged in EPS-stimulated FSHD cells (Supplemental Fig. S3; Supplemental Table S2). In contrast, the categories related to mitosis/cell cycle being exclusive to FSHD-upregulated genes (Fig. 1E, brown), which are unchanged by EPS in control cells (Supplemental Fig. S3). For example, several Z disc/sarcomere-related genes (e.g., TTN^29,30^ and FHL1 ^31,32^) were found to be significantly upregulated in control cells in response to EPS, but not in FSHD cells (Fig. 1C). The ontology categories for downregulated genes are also distinct between control and FSHD groups (Fig. 1E, dark blue and light blue, respectively) (Supplemental Fig. S3; Supplemental Table S2). The results reveal aberrant gene expression responses of FSHD myocytes to EPS, including the failure to upregulate muscle structural and contractile genes normally observed in control myocytes.

### EPS-induced contractility activation is impaired in FSHD myocytes

**Fig. 2:**
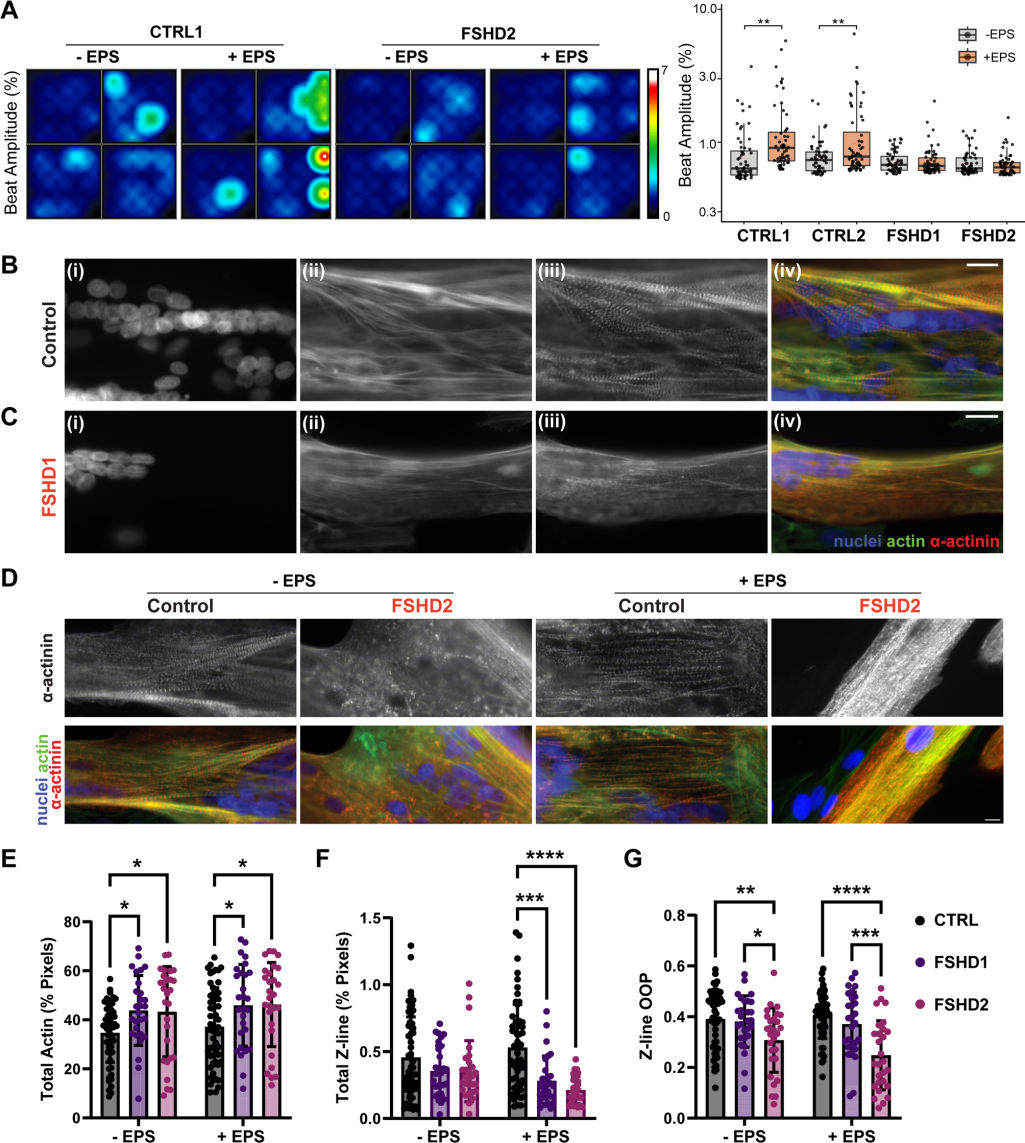
Structural analysis of Control and FSHD skeletal myotubes with and without EPS. (**A**) EPS enhances contractility in control myotubes but not in FSHD myotubes. Beat amplitude (%) was quantified using multielectrode array (MEA) analysis in control and FSHD myotubes, with or without 12-hour electrical pulse stimulation (EPS). Left: Representative heat maps showing beat amplitude (%) in CTRL1 and FSHD2 myotubes under the indicated conditions. Right: Each dot represents the beat amplitude (%) recorded from a single electrode (*n* = 60 electrodes per group). Data are visualized as dot-box plots, presenting the median ± interquartile range (IQR), with mean values indicated by red bars. **p < 0.01, unpaired Student’s t-test. Immunofluorescent images of myotubes from (**B**) Control and (**C**) FSHD1 cell lines stained for (i) nuclei, (ii) actin, and (iii) ɑ-actinin, which are blue, green, and red in (iv) merged image, respectively. (**D**) Immunofluorescent images of myotubes from Control and FSHD2 cell lines under non-paced (-EPS) and paced (+ EPS) conditions. Scale bar = 20 μm. (**E**-**G**) Quantitative analysis of images with bars representing mean and error bars of standard deviation. Significance bars were generated using two-way ANOVA and post hoc Tukey’s Honest Significant Difference (HSD) test, with ***p < 0.001, ** *p* < 0.01, and * *p* < 0.05; (**E**) Percent of image area positive for actin stain; (**F**) Z-line density within cells; (**G**) Z-line orientational order parameter.

To investigate the consequences of the impaired gene expression response to EPS in patient cells, we employed Microelectrode Array (MEA) systems (Axion) to test muscle activation in response to EPS. MEAs enable impedance-based monitoring of contractile activity by analyzing cell–microelectrode interactions, with beat amplitude serving as a key parameter of contractile function^33^. Although control and FSHD myotubes were comparable at the start of the experiment, the mean beat amplitude percentage increased in response to EPS in control cells, but not in FSHD cells (Fig. 2A). The results indicate that EPS fails to promote contractile machinery development in FSHD myotubes.

### Sarcomere structural deviation of patient cells is more apparent after EPS

We next performed quantitative analyses of the structural and morphological characteristics of control and patient striated myotubes with and without EPS by immunostaining using antibodies specific for actin, a-actinin, and DAPI (Fig. 2B-G). Myoblasts were seeded on PDMS and Geltrex coated coverslips and were induced to be differentiated into myotubes (see the methods section). These cells were then subjected to EPS described above followed by fixation, immunostaining, and imaging. Individual myotubes on the coverslips were imaged in a field of view slightly larger than 35,000 µm^2^ (Fig. 2B-D). The higher magnification images (Fig. 2B-D) allowed for an analysis of cytoskeleton structure on the scale of individual myotubes, but it is worth noting that the control lines tended to have more myotubes overall (Supplemental Fig. S2C). We used an automated image analysis tool “ZlineDetection”, which utilizes immunofluorescent staining images of actin (filaments) and α-actinin, a representative Z-line protein, to compute sarcomeric architecture^34^. While α-actinin is myotube-specific, actin is present in both myotubes and undifferentiated mononucleocytes. Therefore, the amount of total actin is affected by the cell density in the field of view. This does not interfere with our sarcomere analysis as ZlineDetection specifically computes sarcomeric Z-line organization in myotubes in which both actin filament and α-actinin must be present (Fig. 2B-D; Supplemental Fig. S2C).

Both patient (FSHD1 and FSHD2) samples have slightly higher total actin than the control under both non-paced and paced conditions (Fig. 2E), which may reflect seeding variability and resulting cell density differences in the field of view (see above). There is no statistically significant difference of the total z-lines between control and patient samples without EPS though there is a slight downward trend in the patient group (Fig. 2F, left). Strikingly, EPS causes the amount of z-lines in FSHD samples to significantly decrease compared to control samples (Fig. 2E, right). This significance is driven by both a slight increase in control z-line content and a decrease in z-line density within the patient lines following EPS. An important measure of striated muscle maturity is the organization of z-lines, which can be measured using the orientational order parameter (OOP)^35^. In the absence of EPS, z-lines in FSHD2 are significantly less aligned than those in both control and FSHD1 samples, which exhibit a similar degree of alignment (Fig. 2G, left). Interestingly, this difference is magnified, and becomes more statistically significant with EPS (Fig. 2G, right). This trend is driven by a slight increase in z-line orientational order of the control, basically no change in FSHD1, and a decrease in the alignment of FSHD2. This implies that EPS is beneficial to the muscle structure of the control, but is either neutral or detrimental to FSHD.

### EPS-induced Z-line disorganization in isogenic FSHD mutant myotubes

**Fig. 3:**
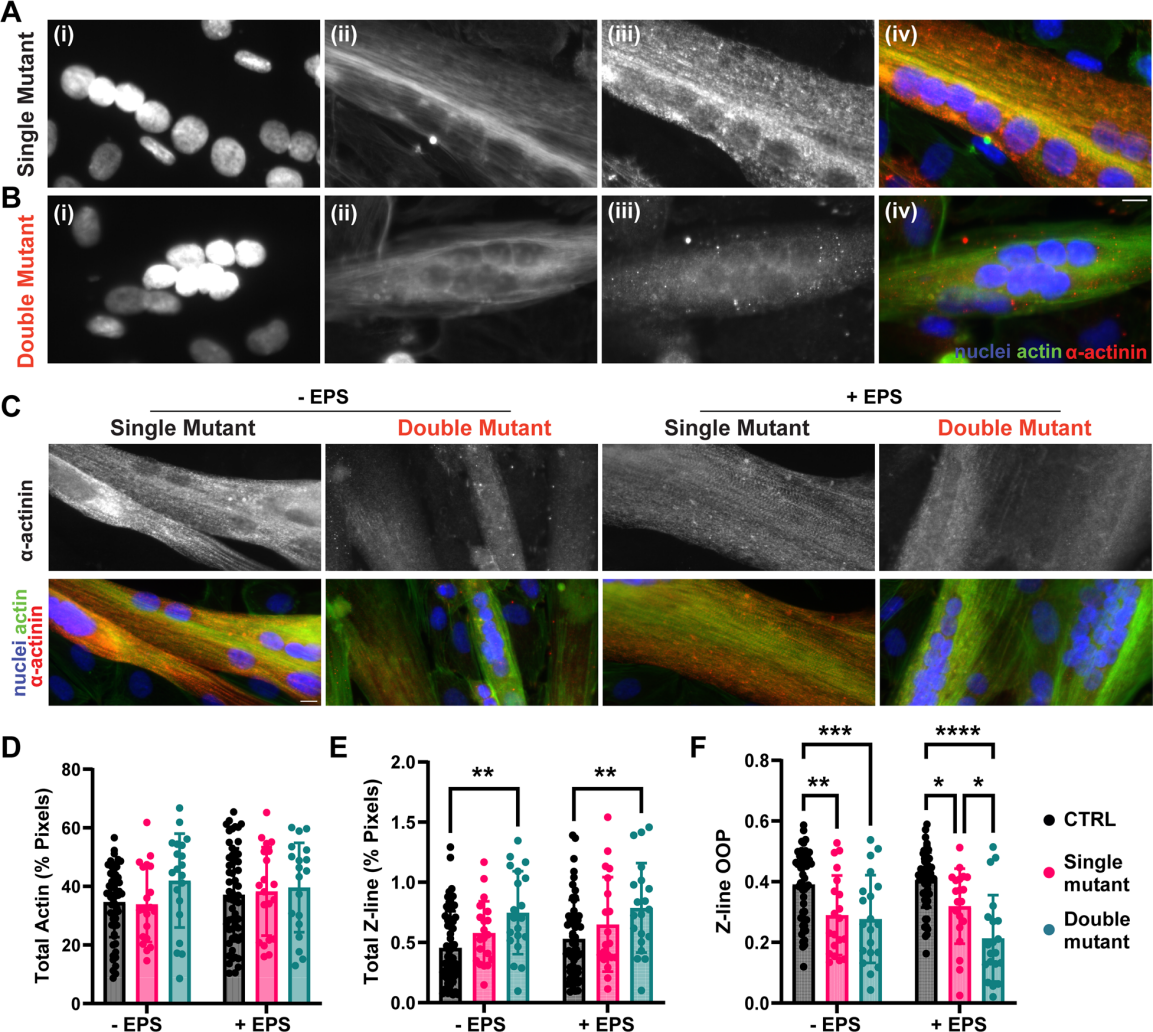
Structural analysis of isogenic single and double mutant myotubes with and without EPS. Immunofluorescent images of myotubes from (**A**) Single Mutant and (**B**) Double Mutant cell lines stained for (i) nuclei, (ii) actin, and (iii) ɑ-actinin, which are blue, green, and red in (iv) merged image, respectively. (**C**) Immunofluorescent images of myotubes with (+ EPS) and without (-EPS) pacing. D-F. Quantitative analysis of images with bars representing mean and error bars of standard deviation. Scale bar = 20 μm. Significance bars were generated using two-way ANOVA and post hoc Tukey’s Honest Significant Difference (HSD) test, with ***p < 0.001, ** *p* < 0.01, and * *p* < 0.05. (**D**) Percent of image area positive for actin stain; (**E**) Z-line density within cells; (**F**) Z-line orientational order parameter.

While we observed differences between control and FSHD patient myocytes, these may come from individual variability. Thus, we used isogenic single (SM) and double (DM) mutant myocytes that harbor *D4Z4* repeat contraction without or with additional *SMCHD1* disease severity modifier gene mutation, respectively, which were derived from the CTRL1 cell line^11^. As previously described^11^, introduction of *D4Z4* contraction alone in a healthy adult myoblast line is not sufficient to establish robust DUX4 gene network activation while having the second mutation in *SMCHD1* gene synergistically increased DUX4 gene network activation (supplemental Fig. S1B)^11^. Using these cell lines, we performed the structural analyses as described above (Figs. 2 and 3A-C). There were no significant differences observed in actin content between the control and isogenic mutant samples irrespective of EPS (Fig. 3D). However, the total amount of z-lines was greater in the double mutant samples than in the control (Fig. 3E). This implies that the formation of z-lines was more efficient in the double mutant, indicating that DUX4-activating mutations do not affect the amount of myotube striation self-assembled during differentiation and maturation. It was more striking, therefore, that the z-lines in single mutant and double mutant samples were significantly less aligned than those in the control without EPS (Fig. 3F, left). With EPS, the control z-lines became much more organized and the single mutant become a little more organized, while the double mutant was less organized. As a result, the difference between the organization of the single mutant and the control became less significant, while the difference between the control and the double mutant becomes even more robust (Fig. 3F, right). This suggests that while the mutations may not directly affect sarcomere striations, they negatively affect EPS-sensitive structural integrity and organization of sarcomeres.

### FSHD is defective in OSTN (musclin) induction

**Fig. 4:**
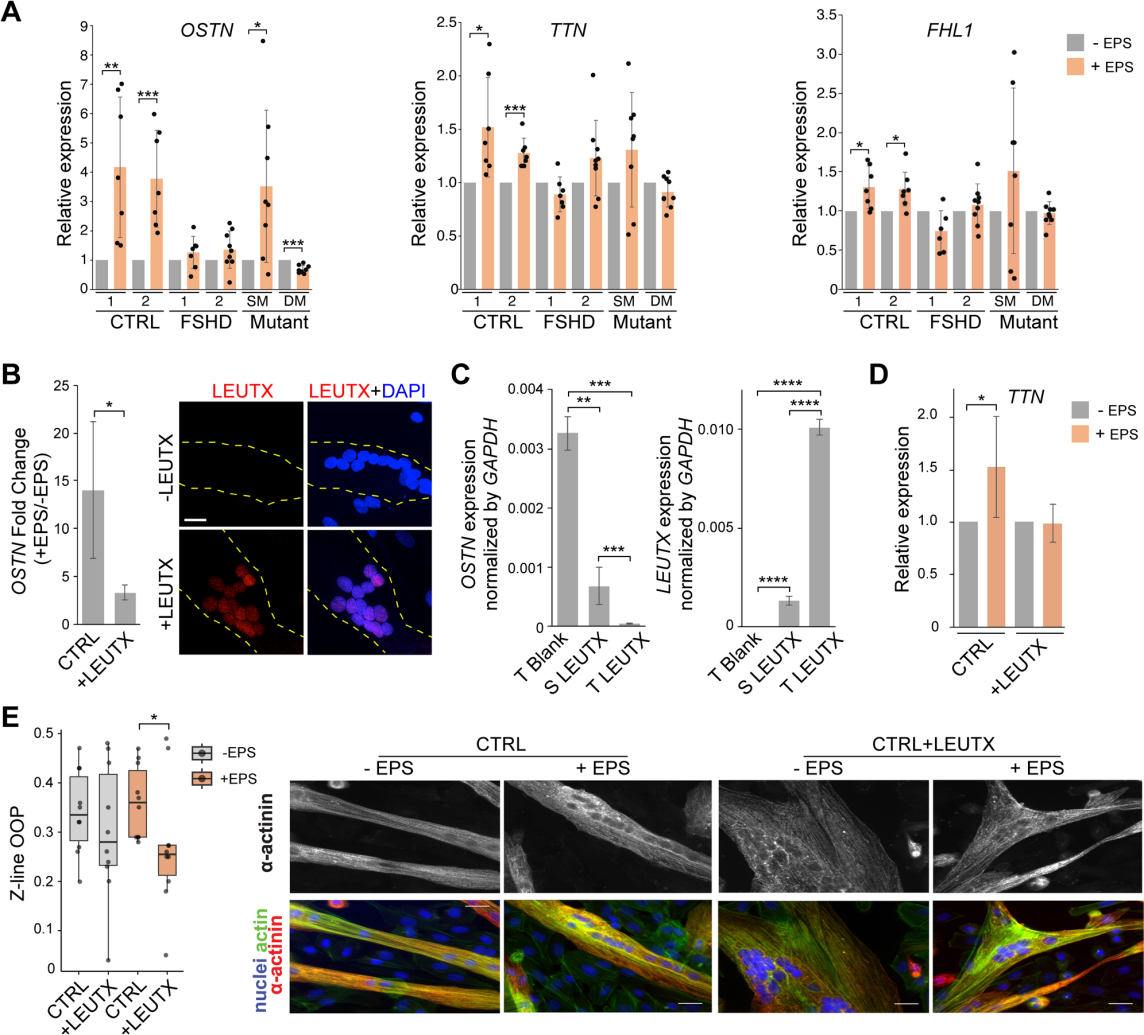
DUX4 gene network activation suppresses EPS-responsive myo-protective gene upregulation. (**A**) Examples of muscle contractile and myo-protective myokine genes that are upregulated in control cells but not in patient and mutant cells. TTN, FHL1 and OSTN are selected based on RNA-seq results for validation by RT-qPCR in control, FSHD, single mutant (SM) and double mutant (DM) cell lines. Gene expression levels are normalized to GAPDH and compared to the corresponding gene expression value in the same cell line without EPS treatment. Data are presented as relative expression (mean ± standard deviation (SD)). Experiments were performed at least 7 times. *p < 0.05, **p < 0.01, and ***p < 0.001 by unpaired Student’s t-test. (**B**) Left: Under DOX induction, the fold change of *OSTN* expression in response to EPS is measured in CTRL1 cells (CTRL) or CTRL1 cells carrying DOX-inducible LEUTX (+ LEUTX). Data are presented as relative expression (mean ± standard deviation (SD)). Experiments are performed 4 times. *p < 0.05, by unpaired Student’s t-test. Right: Examples of LEUTX protein expression in CTRL1 with or without carrying DOX-inducible LEUTX. Cells were fixed after 48 h of DOX treatment starting from differentiation day 8. LEUTX (red) and DAPI (blue). The boundaries of the myotubes are indicated by yellow dashed lines. Scale bar 20 μm. (**C**) Under DOX induction, *OSTN* (left) and *LEUTX* (right) expression in control cells stably carrying DOX-inducible LEUTX (S LEUTX), transiently transduced with DOX-inducible LEUTX (T LEUTX), or transiently transduced with a blank vector (T blank). Data are presented as mean ± standard deviation (SD). Experiments were performed 4 times. **p < 0.01, ***p < 0.001, and ****p < 0.0001 by unpaired Student’s t-test. (**D**) *TTN* expression under DOX induction in CTRL1 cells (CTRL) and CTRL1 cells carrying DOX-inducible LEUTX (+ LEUTX), with or without EPS treatment as indicated. Gene expression levels are normalized to *GAPDH*, and are presented as mean ± standard deviation (SD). Experiments were performed 4 times. *p < 0.05, by unpaired Student’s t-test. (**E**) The effect of LEUTX OE on sarcomere structure. Left: Z-line orientational order parameter analysis of CTRL1 cells (CTRL) and CTRL1 cells carrying DOX-inducible LEUTX (+ LEUTX) under DOX induction, with or without electrical pulse stimulation (EPS). Data are shown as dot-box plots, with individual data points overlaid on boxplots representing the median and interquartile range (IQR). *p < 0.05, as determined by the Wilcoxon rank-sum test. Right: Representative immunofluorescent images of the indicated cells stained for actin and α-actinin, used for Z-line OOP analysis. Scale bar = 25 μm.

The above EPS-sensitive structural defect is accompanied by failure of both patient and mutant cells to upregulate *TTN* and *FHL1* as confirmed by RT-qPCR (Figs. 1C and 4A). The Titin protein encoded by *TTN* is a structural constituent of muscle and plays a critical role in sarcomere elasticity and the regulation of muscle contraction^29,30^. The *FHL1* product, Four and a-Half LIM domains protein 1 (FHL1), localizes to sarcomere and sarcolemma, and regulates sarcomere architecture and assembly. Interestingly, we also found that a myokine gene, *osteocrin (OSTN)* (also known as *musclin*), was even more robustly upregulated in both control samples than *TTN* and *FHL1*, but remained significantly low in FSHD patient and double mutant samples (Fig. 4A). Failed induction of *musclin/OSTN* was further confirmed in additional 2 FSHD1 and 1 FSHD2 cell lines (Supplemental Fig. S4). Musclin is a myokine known to be produced in response to exercise and regulate muscle metabolism and inflammation contributing to muscle endurance and protection^29,36,37^. In single mutant (SM) cells with poor DUX4 target activation, *OSTN* is induced in response to EPS similar to control cells (Fig. 4A; Supplemental Fig. S1B)^11^. Furthermore, although not statistically significant, the expression of *TTN* and *FHL1* tends to be higher and more variable with EPS in SM cells. The results suggest an inverse correlation between DUX4 target gene expression and EPS-stimulated muscle-related gene expression^11^. Taken together, our EPS condition effectively activates exercise-induced musclin/OSTN in healthy control myocytes, which is impeded in FSHD patient/mutant myocytes.

### LEUTX overexpression is sufficient to compromise EPS response in control cells

*LEUTX* is a major DUX4 target transcription factor and plays a significant role in enhancing other DUX4 target gene expression^11^. Importantly, we previously observed significant negative correlation of muscle-related gene expression with LEUTX expression in patient/mutant myotubes by MERFISH^20^. Since the observed gene expression and structural phenotypes appear to correlate with DUX4 target gene expression, we examined whether LEUTX expression can recapitulate these phenotypes in control cells, using a stable control cell line with DOX-inducible LEUTX (Fig. 4B). Because of the low-level leaky expression even in the absence of DOX (Supplemental Fig. S5A), the experiments are performed in the presence of DOX (+ LEUTX) comparing to the parental cell line (CTRL) with DOX. LEUTX protein expression and its nuclear localization in myotubes are confirmed (Fig. 4B; Supplemental Fig. S5B). The results indicate the impaired induction of *OSTN* upon EPS in the presence of LEUTX (Fig. 4B). Comparing the stable cell line and transient lentiviral transduction of LEUTX, which can achieve higher LEUTX expression (Fig. 4C right, S LEUTX and T LEUTX, respectively), we found that the LEUTX expression levels appear to inversely correlate with *OSTN* induction (Fig. 4C). Importantly, overexpression of LEUTX does not induce DUX4 expression, further confirming that LEUTX is downstream of DUX4 and that the expression of LEUTX is sufficient to recapitulate the OSTN suppression phenotype observed in patient and mutant cells (Fig. 4A and B; Supplemental Fig. S1C). Consistent with this, EPS-stimulated TTN induction is also blocked by LEUTX expression (Fig. 4D).

There is a slight decrease of z-line OOP without EPS in the presence of LEUTX, but is not statistically significant (Fig. 4E, left, gray). With EPS, however, the alignment of z-lines in myotubes expressing LEUTX deteriorates significantly compared to the control (CTRL) (Fig. 4E, orange). Thus, LEUTX expression alone in control cells is sufficient to recapitulate EPS-accentuated deterioration of Z-line organization as observed in FSHD patient and double mutant myocytes (Figs. 2F, 3F and 4E). Taken together, our results strongly suggest that DUX4 gene network activation in FSHD contributes to defective EPS-induced muscle protective gene expression and EPS-enhanced disorganization of the muscle sarcomere structure.

## Discussion

While multiple signaling and gene network pathways have been shown to be altered upon DUX4 induction, how they relate to the functionality of the FSHD myocytes was unclear. Our study addressed functional defect of FSHD myocytes at late myotube stage in response to electrical stimulation in vitro. Expression of the major downstream DUX4 target LEUTX gene is sufficient to recapitulate the phenotype, supporting that the observed cell autonomous functional defect is provoked by the DUX4 gene network activation.

We applied high-resolution quantitative measurement of sarcomere structure and organization, and found organizational issues with both FSHD and double mutant subject to EPS as indicated by decreased Z-line OOP. Disorganization of sarcomeres at these length-scales will have two important consequences. First, the amount of force generated by the muscle in the direction of muscle force activation will be significantly reduced^38^. This is in agreement with a previous study using isolated patient muscle fibers demonstrating force reduction (sarcomere dysfunction) in FSHD, particularly in type II muscle fibers, which contributes to muscle weakness^39^. Second, there are likely to be gene expression changes triggered by the change in local orientation of the sarcomeres^38,40^. This is consistent with the observed deficient transcriptomic response of patient and mutant cells to EPS in the current study.

Comparison of double and single mutant cells has been instrumental in demonstrating the synergistic effects of D4Z4 contraction and SMCHD1 mutation on the endogenous DUX4-dependent target gene activation^11^ and allowing us to dissect cellular and transcriptomic changes in the isogenic background with the healthy (parental) control cells. A significant difference in DUX4 target gene expression between single and double mutant cells illuminated the inverse relationship between the level of DUX4 target expression and the adaptive response (both structural and transcriptomic) to EPS. Because the CRISPR mutations were introduced in the adult myoblast stage, however, the effect of FSHD mutations in earlier development is not captured, which may also contribute to the disease pathogenesis and may explain the observed difference of total Z-line (myotube formation) compared to patient cells. The amount of total Z-line in mutant cells is comparable to that of the isogenic parent cells, indicating that FSHD mutations introduced at the adult myoblast stage do not directly affect myotube formation. In contrast, patient myotubes used in our study exhibited lower amounts of total Z-line compared to the controls specifically associated with EPS treatment, suggesting that they are more prone to structural maturation defect in the presence of EPS. It is currently unclear whether this difference is due to variability of individual cell lines or because patient cells have been carrying the FSHD mutations from the embryonic stage, possibly setting up more severe underlying defect. The study is underway to introduce similar mutations in iPSCs to generate isogenic mutant lines, which may address this question.

Previous studies, including our recent results with mutant cells, showed that muscle-related genes tend to be downregulated in differentiated FSHD myotubes even though muscle development in patients and patient myotube differentiation in vitro are not overtly defective^20,21,41–44^. Our current results demonstrated that induction of muscle-related genes, such as *TTN* and *FHL1*, in response to electrical stimuli is impaired in FSHD myocytes. Titin encoded by *TTN* plays a crucial role in sarcomere organization, regulation of muscle contraction, hypertrophic signaling, exercise-induced mechanosignaling, and skeletal muscle remodeling^45^. Moreover, previous studies reported that TTN expression can be upregulated at the transcript or protein level in response to exercise training in various muscle types^46–48^. Lack of adaptive muscle gene response to EPS, therefore, represents cell autonomous defect of FSHD myocytes. It is interesting to note that FHL1 expression was found to be low in FSHD and is inversely correlated to the disease severity^49^. FHL1 mutations were also linked to Emery–Dreifuss-muscular-dystrophy^50^. Furthermore, FHL1 knockout mice develop age-dependent myopathy with myofibrillar/intermyofibrillar disorganization^51^. It is possible that the observed functional deviation of FSHD myocytes exacerbated by EPS may be in some way related to the progressive weakness of muscles in patients as they use their muscle and with age.

We found a significant defect of EPS-induced *musclin/OSTN* myokine gene activation caused by DUX4 target gene activation. While control cells robustly activate *musclin/OSTN* in response to EPS, failure of *musclin/OSTN* induction in response to EPS was observed in multiple FSHD patient cells as well as engineered FSHD mutant cells isogenic to the control cells, strongly suggesting that this is a conserved phenotype associated with FSHD. Importantly, LEUTX overexpression in the absence of DUX4 was sufficient to recapitulate the phenotype, strongly suggesting the critical role of LEUTX in mediating this defective phenotype as a DUX4 downstream target. Interestingly, exercise-induced musclin was recently shown to restrict proliferation of fibro-adipogenic progenitors (FAPs) and facilitate the elimination of apoptotic FAPs, contributing to the benefit of exercise in reduction of fibrosis and fat infiltration^52^. Curiously, prominent FAP expansion and infiltration have been observed in both DUX4-inducible mouse model and patient muscle biopsy samples, which were thought to be a critical contributor of FSHD pathology^53,54^. It is interesting to speculate the deficiency of musclin induction during muscle usage may contribute to the abnormal hyperactivation of FAPs in FSHD progression.

In summary, we found that healthy myotubes exhibit specific adaptive responses to EPS, which is impaired in FSHD patient and mutant myotubes as well as myotubes overexpressing the DUX4 target, LEUTX, indicating the cell autonomous defect of FSHD associated with DUX4 target gene activation. EPS exacerbate disorganization of sarcomeres in conjunction with failure to induce muscle-protective genes, including *musclin/OSTN*, a known exercise-induced myokine gene, providing a new insight into the functional defect and potentially activity-stimulated pathology of FSHD myocytes.

The structural and gene expression phenotype was recapitulated in FSHD mutant cells isogenic to the healthy control cells as well as control cells expressing LEUTX, one of the major DUX4 target genes, linking the phenotype to DUX4 target gene activation. Impaired musclin induction was confirmed in 3 FSHD1 and 2 FSHD2 patient cell lines, which all express significant amount of DUX4 target gene expression. However, patient muscle phenotype and DUX4 target expression are known to be variable, and our recent study suggests that different DUX4 target subpathways may be differentially activated^20^. Thus, it is possible that other pathogenic pathway(s) may differentially influence the patient disease phenotype, contributing to the disease heterogeneity.

## Methods

### Myoblast cell lines

Control1 (or CTRL1), Control2 (or CTRL2), FSHD1, and FSHD2 skeletal myoblast cell lines were immortalized single clones derived from de-identified primary myoblasts obtained from the Cell Repository at University of Rochester Medical Center kindly provided by Dr. Rabi Tawil^11,17,20,21^. Protocols for immortalization and single-clone isolation were described previously^17^. The FSHD1 cell line carries one 4qA allele with 8 *D4Z4* repeats. The FSHD2 cell line carries a heterozygous deletion of 5 bp in exon 10 of the SMCHD1 gene producing a loss of frame with premature stop codon at the deletion (c.1302-1306delTGATA), and 2 4qA alleles with 15 and 19 *D4Z4* repeats. The original two patient myoblasts were selected as representative FSHD cells chosen from > 10 patient cell samples based on their robust DUX4 target gene expression and efficient proliferation and differentiation. The Alt-R CRISPR-Cas9 genome editing system (IDT) was employed to generate single clones of *D4Z4* deletion mutant (DEL) and double mutant (DEL_SM) carrying both SMCHD1 mutation and *D4Z4* deletion as described previously^11^. These mutants are generated from and are isogenic to Control1. Control1, DEL, and DEL_SM were referred to as control, DEL9, and DEL9_SM_A, respectively, in our previous study^11^. The immortalized Control2 line was derived from the primary Control-2 myoblast line^21^. A stable doxycycline (DOX)-inducible LEUTX-overexpressing line was also generated from Control1 (see below). Immortalized skeletal myoblast cells, including control, FSHD, mutant and LEUTX overexpressing cells, were cultured in growth media (high glucose DMEM (Gibco) supplemented with 20% FBS (Omega Scientific, Inc.), 1% Pen-Strep (Gibco), and 2% Ultrasor G (Crescent Chemical Co.).

### Recombinant LEUTX overexpression

The pCW57.1-DUX4-WT lentiviral plasmid (Addgene plasmid #99282) was digested with EcoRI and SalI to remove the DUX4 insert and used as a DOX-inducible lentiviral expression vector. The LEUTX_Flag_PCR primer pair (SI Appendix, Table S3) was used to amplify the human LEUTX ORF from FSHD1 myotube cDNA, and add a C-terminal Flag tag. The PCR product was cloned into the modified vector using the NEBuilder HiFi DNA Assembly Cloning Kit (New England Biolabs) and confirmed by Sanger sequencing (Azenta). Lentiviral particles were produced in 293T cells via co-transfection of pCW57.1-LEUTX or empty pCW57.1(Addgene plasmid #41393), PAX2 (Addgene #12260), and VSVG (Addgene #8454). For transient transduction, CTRL1 myoblasts were transduced with the viral supernatant and, the following day, selected with 2 µg/mL puromycin (Sigma) for three days. To generate a stable DOX-inducible LEUTX-overexpressing cell line, single-cell clones were isolated by FACS into 96-well plates. A single cell clone with good proliferation, differentiation and LEUTX expression was selected for downstream experiments. 10ng/mL DOX was added to the culture medium for 48 h on day 8 of differentiation.

### Myoblast differentiation and electric pulse stimulation

12 well tissue culture plates (FALCON, #353043) were coated with 300–450 µg/ml Geltrex (Thermo Fisher Scientific, #A1413202) overnight in a 37 °C humid incubator. Myoblasts were seeded at 5 x 10^5^ cells per well onto the plates in growth media. Four hours later, growth media was replaced with differentiation media (high glucose DMEM medium supplemented with 2% FBS and 1% ITS (Thermo Fisher Scientific) supplement). Fresh differentiation media was changed every day. On day 10 of differentiation, Electrical pulse stimulation (EPS) was applied to the cells using a C-Pace EM culture pacer (IonOptix, Westwood, Ma, USA) applied for 12 h, with 4 ms pulses at 5 V, and a frequency of 1 Hz. Six hours after completion of EPS, cells with or without EPS treatment were harvested for RNA isolation or Immunofluorescent staining. The only exception was the assessment of muscle activation in response to EPS, which was performed using a Microelectrode Array (MEA) system (Axion).

### Multielectrode array (MEA)

3 M Myoblasts were seeded onto 10 cm culture dish in growth media overnight. Then growth media was replaced with differentiation media. On day 6 of differentiation, myotubes were removed by gentle pipetting, leaving mononuclear cells (MNCs) attached to the culture dish. The remaining MNCs were collected and resuspended at a concentration of 2 million cells per mL. A 5 µL droplet of the cell suspension was then carefully replated onto the recording electrode area of each well in a fibronectin-coated CytoView MEA 24-well plate (Axion BioSystems, Inc.). Incubate the MEA plate with the seeded MNCs in a cell culture incubator at 37 °C, 10% CO2 for 1 h for cells to adhere, followed by adding 500 µL differentiation medium. Four days later, the cell culture MEA plate was moved to the Maestro Edge platform for recordings with or without 12 h electric stimulation at 1 Hz, 1200 mV and 500 µs duration time. Cardiac Analysis Tool software AxIS Navigator (Axion BioSystems, Inc.) for beat detection were used to determine the beat amplitudes of the samples. For each sample, data from 120 electrodes across 8 wells were pooled together. The top 50% (i.e., 60 electrodes) with the highest beat amplitudes (%) in each sample were used for graph and data analysis to assess whether EPS improve contractility significantly within the same cell type.

### RNA isolation and quantitative real-time RT-PCR (RT-qPCR)

RNA was extracted using RNeasy Plus Mini kit (Qiagen), and complementary DNA (cDNA) was made using 300–500 ng of total RNA with SensiFAST cDNA Synthesis Kit (Meridian Bio. # BIO-65054) following the manufacturer’s instructions. qPCR was performed by using AzuraView GreenFast qPCR Blue Mix LR (Azura Genomics Inc.). The genes and their corresponding PCR primers are listed in SI Appendix Table S3.

### RNA-seq and data processing

Total RNA was extracted by using the RNeasy kit (QIAGEN). Between 50 and 100 ng of RNA was converted to cDNA using the Smart-Seq 2 protocol (3). DNA libraries were constructed using Nextera DNA Flex Library Prep Kit (Illumina). DNA samples were sequenced on the Illumina NovaSeq platform with around 20 million reads per sample. RNA-seq raw reads were pseudo-aligned to the human reference transcriptome (Homo_sapiens.GRCh38.cdna) using Kallisto (4) (v0.44.0). The Kallisto output files were read into R, and the differential expression analysis was performed by sleuth (v0.30.1). For each cell line, differential expression p-values and Bonferroni corrected q-values were calculated using a likelihood ratio test comparing the samples with EPS treatment to the samples without EPS treatment. Differential genes were determined using the cutoff q-value < 0.1 and absolute beta value > 0.2.

### Cell fixation and immunofluorescent staining

Fixation and immunostaining were performed following established protocols^34^ with slight modifications. Briefly, the cells seeded onto Geltrex coated glass coverslips were fixed in 4% paraformaldehyde (VWR, Radnow, PA, USA) supplemented with 0.05% Triton X-100 (Sigma-Aldrich, Saint Louis, MO, USA) in PBS for 15 min. Each well was washed three times with PBS for and blocked in blocking buffer (0.02% saponin, 0.05% NaN3, 1% BSA, 4% horse/goat serum and 0.1% gelatin in PBS) for 15 min at 37 °C. Coverslips were incubated overnight at 4 °C with either Alexa Fluor 488 Phalloidin (for actin detection; Thermo Fisher, Grand Island, NY, USA) or primary antibodies. After three washes with PBS, samples were incubated with the appropriate fluorescent secondary antibodies for 30 min at 37 °C, followed by another three PBS washes. Nuclei were counterstained with DAPI (Sigma), and the samples were mounted using ProLong™ Diamond Antifade Mountant (Thermo Fisher Scientific). Details of the primary and secondary antibodies are provided in SI Appendix, Table S4.

### Image and data acquisition

The bright-field images were acquired using an IX-81 inverted motorized microscope (Olympus, Center Valley, PA, USA) or a BZX800 microscope (Keyence, Itasca, IL, USA). The immunofluorescence images were acquired with an IX-83 inverted motorized microscope (Olympus America, Center Valley, PA, USA). Images were taken using an UPLFLN 40x oil immersion objective (Olympus America, Center Valley, PA, USA) and a digital CCD camera ORCA-R2 C10600-10B (Hamamatsu Photonics, Shizuoka Prefecture, Japan). 10 different myotubes per coverslip were located and imaged at 40x magnification (6.22 pixels/µm).

### Image analysis

Quantitative image analysis was performed using ZlineDetection, which is available on Github (https://github.com/Cardiovascular-Modeling-Laboratory/zlineDetection*)*^34^. ZlineDetection was implemented in MATLAB version 24.1.0.2603908 (R2024a) (MathWorks, Natick, MA). Parameters were not changed from the recommended as listed and described in the Github repository for ZlineDetection. The total number of actin and z-line pixels were normalized to the total number of pixels in each image. Significance tests were generated using two-way ANOVA. An important aspect of this analysis in respect to this work is that ZlineDetection identifies all the pixels from the alpha-actinin stain that are most likely to be part of the striated z-lines. The code than provides analysis of the architecture metrics like the z-line orientational order parameter (OOP) based purely on the alpha-actinin stain that constitutes the myotube as other structures cannot have striated z-lines or the alpha-actinin staining.

### Western blotting

Cells were lysed in 2X Laemmli Buffer (Bio-Rad) with 5% beta-mercaptoethanol, sonicated, boiled, and separated by 4%–20% TEO-Tricine gel (Abcam). Then the samples were transferred to nitrocellulose membranes, blocked with Pierce Protein-Free T20 Blocking Buffer (Thermo Fisher Scientific), and blotted with the desired antibodies (SI Appendix, Table S4). Horseradish peroxidase-conjugated anti-mouse-IgG (Promega), or anti-rabbit-IgG (Promega) were used as secondary antibodies. Immunoblots were developed with SuperSignal West Pico PLUS Chemiluminescent Substrate (Thermo Fisher Scientific). Images were acquired with Fujifilm LAS-4000 Image Analyzer (GE Healthcare).

### Statistical analyses

Statistical comparisons between two groups were conducted using either a two-tailed Student’s t-test or the Wilcoxon rank-sum test. For comparisons involving multiple groups, two-way ANOVA was performed. Statistical significance was defined as *p* < 0.05. Quantitative data are presented as mean ± standard deviation (SD) or median ± interquartile range (IQR), as appropriate. Details regarding the statistical tests and data presentation used for each dataset are provided in the corresponding figure legends.

## Supplementary Information

Below is the link to the electronic supplementary material.

Supplementary Material 1

Supplementary Material 2

Supplementary Material 3

Supplementary Material 4

## Data Availability

All study data are included in the article and/or supporting information. RNA sequencing data have been deposited (dbGaP: phs002554).
