# Supplementary material for: Adaptive response to electrical pulse stimulation is impaired in FSHD myotubes by DUX4 gene network activation
Source: Sci Rep. 2025 Dec 17;16:2409. doi: 10.1038/s41598-025-32385-0 (PMC12820078; doi:10.1038/s41598-025-32385-0)
Supplement: Supplementary file 3 — Supplementary Material 3 [file 41598_2025_32385_MOESM3_ESM.pdf]

Supplementary Information for

Adaptive response to electrical pulse stimulation is impaired in FSHD myotubes by DUX4 gene network activation

Xiangduo Kong, Ananya Rajagopal, Skylar Renee Foust, Jonovan Osorio, Anna Grosberg, and Kyoko Yokomori

Anna Grosberg
Email: grosberg@uci.edu

Kyoko Yokomori
Email: kyokomor@uci.edu

This PDF file includes:

Fig. S1 *DUX4* and DUX4 target genes expression in control, FSHD and mutant cells.

Fig. S2 Representative images and differentiation efficiency of control, patient, mutant and LETUX-expressing control cell lines.

Fig. S3 Distinct EPS-induced expression changes of genes in the GO term categories in Figure 1E in Control and FSHD cell lines at the late differentiation stage.

Fig. S4 *OSTN* is not upregulated by EPS in multiple FSHD patient cell lines.

Fig. S5 LEUTX expression in DOX-inducible LEUTX stable cells.

Table S3. Primer sequences used in this study.

Table S4. Antibodies used in this study.

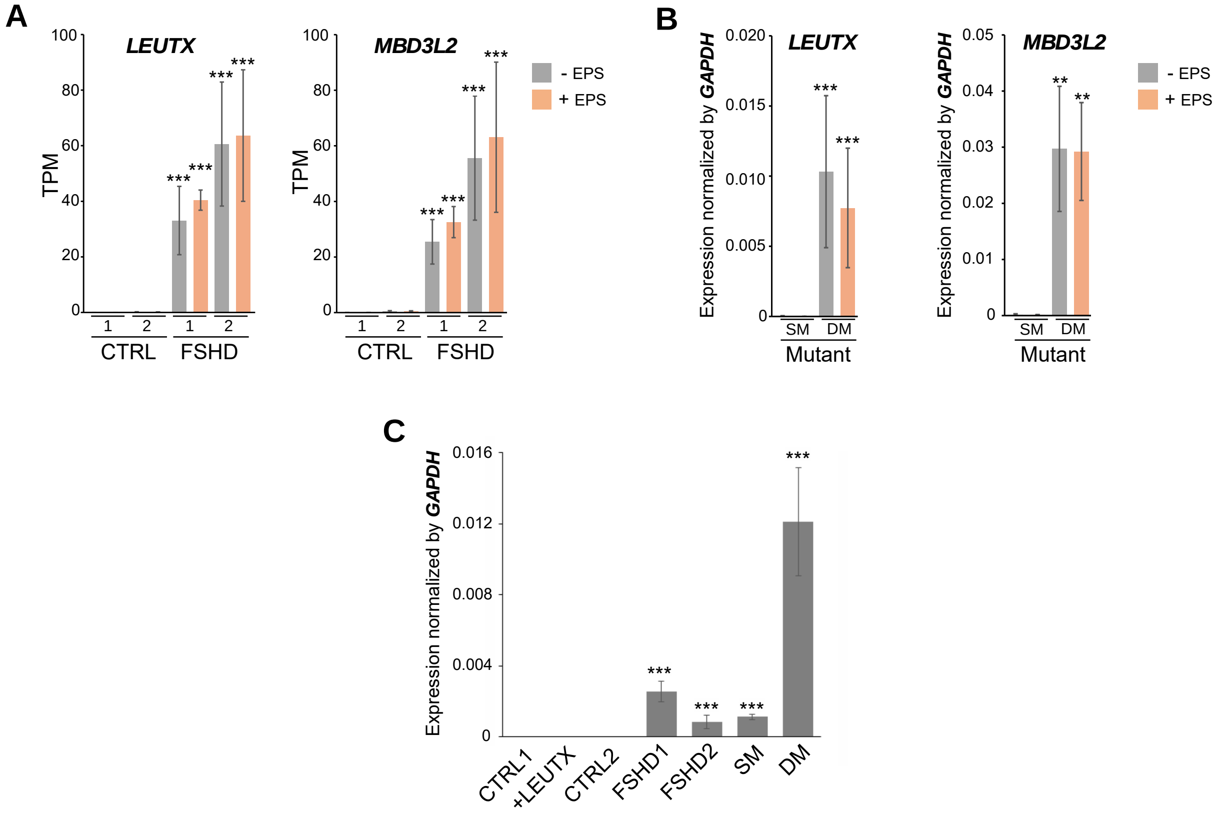

Fig. S1 *DUX4* and DUX4 target genes expression in control, FSHD and mutant cells. (A) Expression of DUX4 target genes *LEUTX* (left) and *MBD3L2*(right) from the RNA-seq results of Control and FSHD samples with or without EPS treatment. Expression values are TPM (Transcripts Per Million). Data are presented as mean ± standard deviation (SD). Experiments were performed in triplicate. ***p < 0.001, compared to control, as determined by unpaired Student's t-test. (B) DUX4 target gene expression levels of *LEUTX* (left) and *MBD3L2* (right) in single mutant (SM) and double mutant (DM) samples, with or without EPS, were determined by RT-qPCR. At least four biological replicates were performed for each sample. Data are presented as mean ± standard deviation (SD). **p < 0.01 and ***p < 0.001, as determined by unpaired Student's t-test. (C) *DUX4* expression levels were measured by RT-qPCR 3. *DUX4* was significantly upregulated in FSHD patient cells and the mutant cell line, but not in LEUTX-overexpressing (OE) cells. *DUX4* RNA expression is not proportional to target gene expression as previously described 1,2. At least three biological replicates were performed for each sample. Data are presented as mean ± standard deviation (SD). ***p < 0.001, as determined by unpaired Student's t-test.

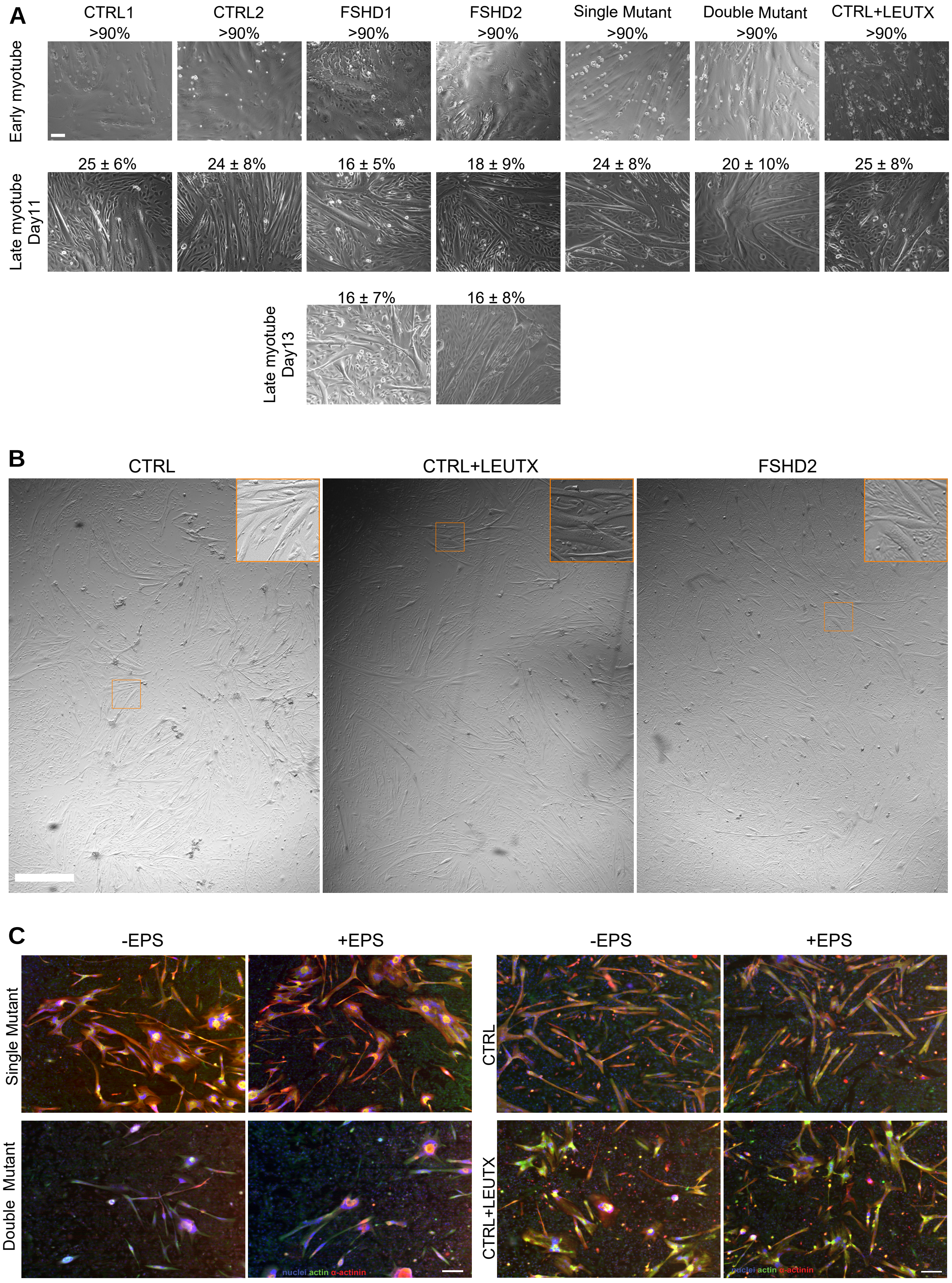
A
CTRL1
>90%
CTRL2
>90%
FSHD1
>90%
FSHD2
>90%
Single Mutant
>90%
Double Mutant
>90%
CTRL+LEUTX
>90%
Early myotube
25 ± 6%
24 ± 8%
16 ± 5%
18 ± 9%
24 ± 8%
20 ± 10%
25 ± 8%
Late myotube
Day11
16 ± 7%
16 ± 8%
Late myotube
Day13
B
CTRL
CTRL+LEUTX
FSHD2
C
-EPS
+EPS
-EPS
+EPS
Single Mutant
Double Mutant
CTRL
CTRL+LEUTX
nuclei actin α-actinin
nuclei actin α-actinin

Fig. S2 Representative images and differentiation efficiency of control, patient, mutant and LETUX-expressing control cell lines. (A) At the early stage (day 4–5), all cell lines showed high fusion efficiency. Subsequently, a large number of myotubes detached, and a second round of proliferation and fusion of mononuclear cells occurred. At the late stage (day 11), fusion efficiency is lower, and myotubes appeared more mature compared with the early stage. Because FSHD cells showed low differentiation efficiency at the late stage, we extended the culture period by two additional days (day 13); however, this did not lead to any improvement in differentiation efficiency, indicating that this is not due to differentiation delay. Control cells with or without LEUTX overexpression exhibited comparable differentiation efficiency. Differentiation efficiency at the late stage was quantified as the percentage of myotube area within each field. Four randomly selected regions were analyzed for each late-stage sample, excluding empty areas caused by myotube detachment. The quantified data are displayed on top of each corresponding image and are presented as mean ± standard deviation (SD). At the late stage, the myotubes are prone to detachment during fixation and staining. Therefore, conventional MyHC staining cannot be used for fusion index calculation. Scale bar 100 μm. (B) Representative low magnification (2X) bright field images show late stage myotubes from control1 (CTRL), LEUTX-overexpressing control1 (CTRL + LEUTX), and FSHD2 cell lines. Scale bar 1000 μm. (C) Representative 4× immunofluorescent staining images of myotubes from Single Mutant, Double Mutant, Control, and LEUTX-overexpressing cell lines under non-paced (−EPS) and paced (+EPS) conditions, showing staining for nuclei (blue), actin (green), and α-actinin (red), as indicated. Detachment and loss of myotubes during washing, fixation and immunostaining procedure were more prominent in double mutant samples with higher DUX4 target expression compared to controls (see original live cell images in (A) and (B)). Scale bar = 200 μm.

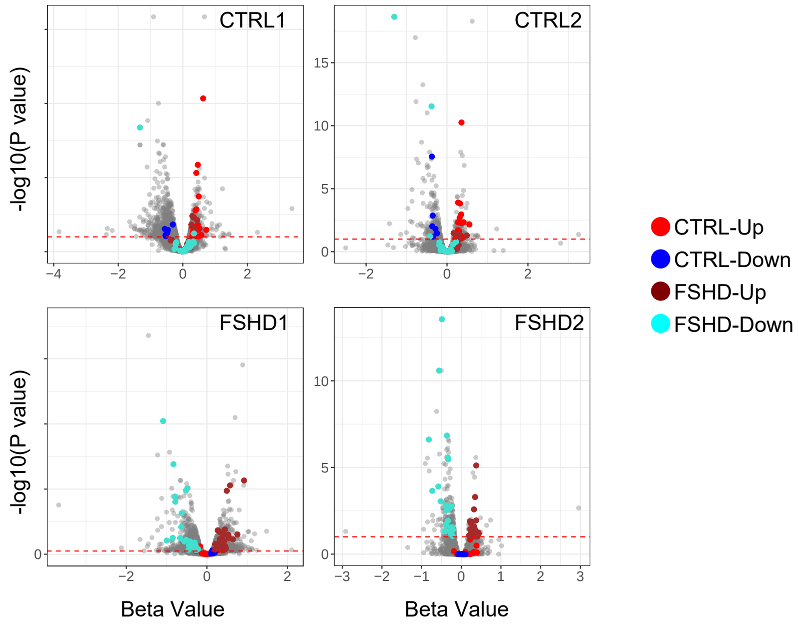

Fig. S3 Distinct EPS-induced expression changes of genes in the GO term categories in Figure 1E in Control and FSHD cell lines at the late differentiation stage. The genes found in the GO term enrichment analysis in Figure 1E are visualized in the volcano plots in Figure 1C. Volcano plots show gene expression differences between EPS-treated and untreated conditions in each cell line at the late differentiation stage. Enriched GO terms of differentially expressed genes in response to EPS in Control and FSHD lines are different, and corresponding genes exhibit distinct expression patterns. Same color scheme was used as in Figure 1E for genes in each group as indicated.

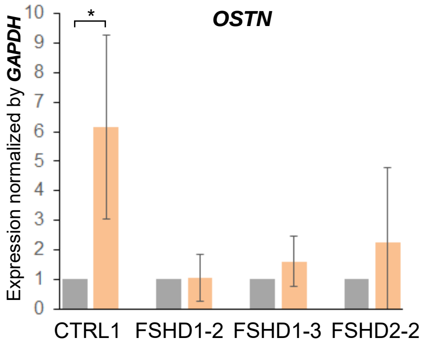

Fig. S4 *OSTN* is not upregulated by EPS in multiple FSHD patient cell lines. *OSTN* expression, with and without EPS treatment, was examined by RT-qPCR in 3 additional FSHD cell lines (two FSHD1 and one FSHD2) as indicated [1,2]. The FSHD1-2 cell line carries a single 4qA allele with 6 D4Z4 repeats. The FSHD1-3 cell line carries two 4qA alleles with 2 and 22 D4Z4 repeats, respectively. The FSHD2-2 cell line carries a heterozygous c.4267C>T mutation in the SMCHD1 gene, resulting in a premature stop codon at amino acid 1423 (of 2005), and possesses one 4qA allele with 13 D4Z4 repeats. CTRL1 served as a positive control confirming the efficient induction of *OSTN* by EPS. Gene expression levels were normalized to *GAPDH* and compared to the corresponding untreated samples of the same cell line. Results are presented as relative expression (mean ± SD) from at least three independent experiments.
* $p < 0.05$, unpaired Student's t-test.

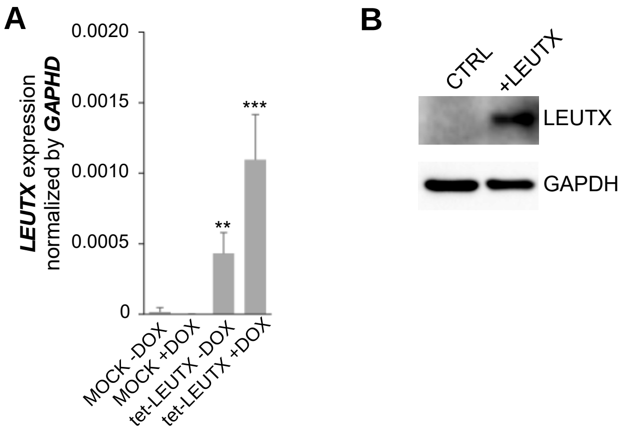

Fig. S5 LEUTX expression in DOX-inducible LEUTX stable cells. (A) Control cells and control cells stably expressing DOX-inducible LEUTX were treated with or without DOX for 48 hours, starting from differentiation day 8. Cells were harvested for *LEUTX* RT-qPCR analysis 18 hours after DOX removal. Each condition was analyzed with at least three biological replicates. Data are presented as mean ± standard deviation (SD). $^{**}p < 0.01$ and $^{***}p < 0.001$, unpaired Student's t-test. (B) Western blot analysis of LEUTX overexpression in CTRL1 cells and CTRL1 cells stably carrying DOX-inducible LEUTX under DOX induction. The LEUTX band was detected using an anti-LEUTX antibody (15 s exposure). After stripping, the same membrane was reprobed with an anti-GAPDH antibody as a loading control (2 s exposure). LEUTX and GAPDH panels are cropped from different regions of the same membrane.

Table S3. Primer sequences used in this study.

PCR primers	
LEUTX_Flag_PCR _F	5'-GTGGATCCCCCGGGCTGCAGGAATTCGCCACCATGTTT GAAGGGCCAAGGCGTTATCGTCGGC-3'
LEUTX_Flag_PCR _R	5'-ATCCAGTCACTATGGTCGAGGTCGACTTACTTGTCGTCA TCGTCTTTGTAGTCCACTGAAGATTGGAGCTGGTCA-3'
Gene expression RT-qPCR primers	
*DUX4*_qPCR_F	5′-CCCAGGTACCAGCAGACC-3′
*DUX4*_qPCR_R	5′-TCCAGGAGATGTAACTCTAATCCA-3′
*LEUTX*_qPCR_F	5'-GGGAAACTGGCTTCAAAGCTA-3'
*LEUTX*_qPCR_R	5'-TGATGGCCGTGTCTGCATTT-3'
*MBD3L2*_qPCR_F	5'-GCGTTCACCTCTTTTCCAAG -3'
*MBD3L2*_qPCR_R	5'-GCCATGTGGATTTCTCGTTT -3'
*GAPDH*_qPCR_F	5'-TCGACAGTCAGCCGCATCT-3'
*GAPDH*_qPCR_R	5'-CCGTTGACTCCGACCTTCA-3'
*OSTN*_qPCR_F	5'-ACTGACCTGACAGCAAAACTC-3'
*OSTN*_qPCR_R	5'-CCTTTGTGATCTACAGAGCCAG-3'
*TTN*_qPCR_F	5'-CCCCATCGCCCATAAGACAC-3'
*TTN*_qPCR_R	5'-CCACGTAGCCCTCTTGCTTC-3'
*FHL1*_qPCR_F	5'-TGCTGCCTGAAATGCTTTGAC-3'
*FHL1*_qPCR_R	5'-GCCAGAAGCGGTTCTTATAGTG-3'

Table S4. Antibodies used in this study.

Antibodies	SOURCE	IDENTIFIER
Rabbit polyclonal anti-LEUTX (1:2000)	Thermofisher	Catalog number PA5-59595; RRID:AB_2643351
Mouse Monoclonal anti-GAPDH (1:2000)	GeneTex	Catalog number GTX627408, RRID:AB_11174761
Mouse Monoclonal Anti-α-Actinin antibody (1:200)	Sigma	Catalog number A7811
Alexa Fluor 594 goat anti-mouse IgG (1:200)	Thermofisher	Catalog number A-11005
Alexa 555 Donkey anti-Rabbit IgG (1:1000)	Thermofisher	Catalog number A31572; RRID:AB_162543
Anti-Rabbit IgG, HRP linked (1:10000)	Promega	Catalog number4018; RRID:AB_430833
Anti-Mouse IgG, HRP linked (1:10000)	Promega	Catalog number4028; RRID:AB_430834

References

1 Chau, J. *et al*. Relationship of DUX4 and target gene expression in FSHD myocytes. *Hum. Mutat.* **42**, 421-433. (2021).

2 Kong, X. *et al*. Engineered FSHD mutations results in D4Z4 heterochromatin disruption and feedforward DUX4 network activation. *iScience* **27**, 109357, doi:10.1016/j.isci.2024.109357 (2024).

3 Krom, Y. D. *et al*. Generation of Isogenic D4Z4 Contracted and Noncontracted Immortal Muscle Cell Clones from a Mosaic Patient: A Cellular Model for FSHD. *Am. J. Pathol.* **181**, 1387-1401. (2012).
