# Supplementary material for: Adaptive response to electrical pulse stimulation is impaired in FSHD myotubes by DUX4 gene network activation
Source: Sci Rep. 2025 Dec 17;16:2409. doi: 10.1038/s41598-025-32385-0 (PMC12820078; doi:10.1038/s41598-025-32385-0)
Supplement: Supplementary file 4 — Supplementary Material 4 [file 41598_2025_32385_MOESM4_ESM.pdf]

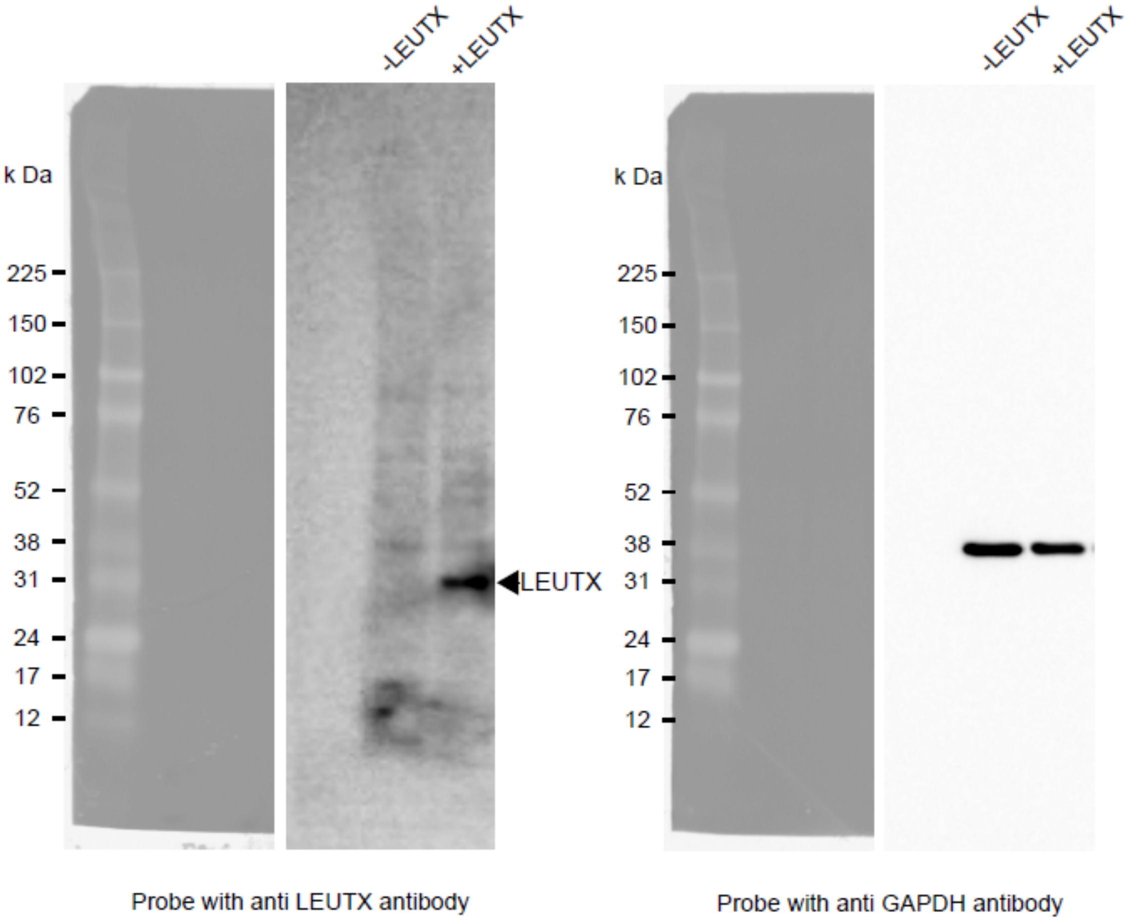

Full-length Western blot images corresponding to Figure S5B. The membrane was first probed with anti-LEUTX (left panel), then stripped and reprobed with anti-GAPDH (right panel). Molecular weight markers are shown on the left of each blot, and the arrowhead indicates the position of LEUTX.
